# Requirement of miR-9-dependent regulation of Myocd in PASMCs phenotypic modulation and proliferation induced by hepatopulmonary syndrome rat serum

**DOI:** 10.1111/jcmm.12631

**Published:** 2015-07-06

**Authors:** Duo Xu, Jian-teng Gu, Bin Yi, Lin Chen, Guan-song Wang, Gui-sheng Qian, Kai-zhi Lu

**Affiliations:** a Department of Anaesthesia, Southwest Hospital, Third Military Medical UniversityChongqing, China; b Institute of Respiratory Disease, Xinqiao Hospital, Third Military Medical UniversityChongqing, China

**Keywords:** hepatopulmonary syndrome, microRNA, Myocd, pulmonary artery smooth muscle cells

## Abstract

Hepatopulmonary syndrome (HPS) is characterized by a triad of severe liver disease, intrapulmonary vascular dilation and hypoxaemia. Pulmonary vascular remodelling (PVR) is a key feature of HPS pathology. Our previous studies have established the role of the pulmonary artery smooth muscle cell (PASMC) phenotypic modulation and proliferation in HPS-associated PVR. Myocardin, a robust transcriptional coactivator of serum response factor, plays a critical role in the vascular smooth muscle cell phenotypic switch. However, the mechanism regulating myocardin upstream signalling remains unclear. In this study, treatment of rat PASMCs with serum drawn from common bile duct ligation rats, which model symptoms of HPS, resulted in a significant increase in miR-9 expression correlated with a decrease in expression of myocardin and the phenotypic markers SM-α-actin and smooth muscle-specific myosin heavy chain (SM-MHC). Furthermore, miRNA functional analysis and luciferase reporter assay demonstrated that miR-9 effectively regulated myocardin expression by directly binding to its 3′-untranslated region. Both the knockdown of miR-9 and overexpression of myocardin effectively attenuated the HPS rat serum-induced phenotype switch and proliferation of PASMCs. Taken together, the findings of our present study demonstrate that miR-9 is required in HPS rat serum-induced phenotypic modulation and proliferation of PASMCs for targeting of myocardin and that miR-9 may serve as a potential therapeutic target in HPS.

## Introduction

Hepatopulmonary syndrome (HPS) is a life-threatening complication of liver disease characterized by a triad of advanced liver disease, intrapulmonary vascular dilation (IPVD) and arterial hypoxaemia 1,2. The syndrome is a severe pulmonary sequel, which can affect 5–32% of patients with cirrhotic liver disease and has been associated with poorer survival rate 3. To date, there is no effective treatment for HPS other than liver transplantation. Our previous research demonstrated that HPS rat serum induced phenotypic modulation and excessive proliferation of pulmonary artery smooth muscle cells (PASMCs) 4,5, which have been identified as two major pathophysiological characteristics in HPS-associated pulmonary vascular remodelling (PVR).

Myocardin, a robust transcriptional coactivator of serum response factor (SRF), is able to stimulate the expression of vascular smooth muscle cell (VSMC) marker genes and inhibit the cell cycle 6–8. It has been reported that myocardin is expressed in the heart and in most developing and adult SMC compartments. Several lines of evidence suggest that myocardin plays a pivotal role in PVR and various other proliferative vascular diseases 9–11.

MicroRNAs (miRNAs) are small non-coding RNAs that negatively regulate gene expression *via* degradation or translational inhibition of protein-encoding mRNAs 12–14. Such posttranscriptional regulation affects various biological processes, including cell proliferation and differentiation and cell type-specific function, and is involved in several cardiovascular diseases 15–17. Since the first report on the role of miRNA in VSMCs was published in 2007, miRNAs such as miR-143/145, miR-21, and miR-20a have been shown to regulate various aspects of VSMC biology 18–21. However, it is not yet clear which miRNAs regulate myocardin and whether targeting myocardin by miRNAs can regulate HPS-associated PVR.

In this study, we investigated the effect of HPS rat serum on miRNAs predicted to target myocardin and identified miR-9 as the most significantly up-regulated miRNA in cultured PASMCs. We also determined the potential regulatory role of miR-9 in regulating myocardin expression and the HPS rat serum-induced phenotypic modulation and excessive proliferation of PASMCs.

## Materials and methods

### Animal model

All procedures performed on the rats were conducted according to the guidelines from the National Institutes of Health. An experimental HPS rat model was successfully established by common bile duct ligation (CBDL), as described in our previous study 5,22. A total of 60 male Sprague–Dawley rats (180–220 g, 6 weeks), which were purchased from the Laboratory Animal Center of Third Military Medical University, were used in this study. The experimental group (*n* = 30) underwent CBDL. The control group (*n* = 30) underwent a sham operation. The animals were studied 5 weeks after the operation. The assessment of HPS in CBDL rats was based on the following criteria: gas exchange dysfunction (PaO_2_ <85 mmHg, A-aDO_2_ >18 mmHg) and confirmation of IPVD by histopathological analysis 4,22. Serum was separated from sham-operation control rats or CBDL rats blood samples (7–8 ml), and then centrifuged at 2000 × g/min. in a Gyria for 10 min. at 4°C. Following filtration with cellulose acetate membranes, the serum was inactivated at 56°C for 30 min. and stored at −80°C for use in subsequent experiments.

### Cell culture

Cultured rat PASMCs used in the entire experiments were isolated from healthy Sprague–Dawley rats, passaged and cultured in DMEM with 10% FBS and used for experiments between passages 4 and 9. For all experiments, PASMCs treated with normal rat serum or HPS rat serum come from the same split cells. The purity and identity of PASMCs was verified by their typical morphological pattern (peak and valley formation) and immunocytochemical staining with an antibody against SM-α-actin, as previously reported 23,24. When the cells reached approximately 80% confluence, the original serum was replaced with 0.1% FBS. Following 24 hrs of synchronous growth, PASMCs were divided into two groups: group C consisted of PASMCs that were cultured in DMEM (4 ml) supplemented with normal rat serum (5%/0.2 ml) and group HPS consisted of PASMCs that were incubated in DMEM (4 ml) containing HPS rat serum (5%/0.2 ml) for various time periods: 24 hrs (T1), 48 hrs (T2) and 72 hrs (T3).

### RNA extraction and quantitative real-Time PCR

Total RNA was extracted from PASMCs using the miRNeasy Mini Kit (Qiagen, Hilden, Germany) and quantified using a NanoDrop spectrophotometer (Thermo Scientific, Waltham, MA, USA). For mature miRNA detection, template RNA (2 μg) was reverse transcribed to cDNA using the miScript II RT Kit with miScript Hispec buffer (Qiagen) followed by real-time PCR using the miScript SYBR Green PCR Kit (Qiagen) with the manufacturer-provided miScript Universal primer. For mRNA expression analysis, template RNA (500 ng) was reversely transcribed to cDNA using the same kit with miScript Hiflex Buffer followed by real-time PCR using miScript SYBR Green PCR Kit (Qiagen) with QuantiTect primer. The results were normalized to U6 for mature miRNAs and to β-actin for myocardin. Data analysis was performed using the comparative Ct method. Myocardin primers were as follows: forward, 5′-GAGAGCACAATTGCACACCAT-3′; reverse, 5′-CTCTGAGACTCGGGCAATC-3′. β-actin primers were as follows: forward, 5′-CATCCGTAAAGACCTCTATGCCAAC-3′; reverse, 5′-ATGGAGCCACCGATCCACA-3′.

### Western blotting

Cultured PASMCs were lysed and subjected to total protein extraction according to the manufacturer’s instructions. Protein concentration was quantified using the bicinchoninic acid (BCA) assay. Equal quantities of protein were separated by SDS-PAGE and then transferred onto polyvinylidene fluoride (PVDF) membranes. Membranes were blocked for 1 hr using 5% skim milk in Tris-Buffered Saline Tween (TBST) at room temperature, and then incubated overnight at 4°C with appropriate primary antibodies (myocardin 1:500, SM-α-actin 1:1000, SM-MHC 1:1000 and β-actin 1:1000; Santa Cruz Biotechnology, Santa Cruz, CA, USA), followed by a 1 hr incubation at room temperature with secondary antibody (HRP-conjugated rabbit anti-goat IgG; 1:10,000; Santa Cruz Biotechnology). Finally, the membranes were stained with diaminobenzidine, scanned and stored using a gel imaging system. The optical density of immunoreactivity was measured and analysed with an Alpha Imager [Protein Simple (San Francisco, CA, USA), CA, USA].

### Cell transfection

Ectopic expression of miR-9 in cultured PASMCs was achieved by transfection with miR-9 mimics or inhibitors (Qiagen). Overexpression of the myocardin gene was performed by transient transfection with MYOCD expression plasmids (provided as a generous gift from the Institute of Respiratory Diseases, Xinqiao Hospital, Third Military Medical University). The efficiency of transfection was confirmed using western blot and real-time PCR.

### Site-directed mutagenesis and Luciferase reporter assay

The 3′-untranslated region (UTR) of MYOCD was amplified by RT-PCR out of genomic DNA. By using the Quick-change Site-Directed Mutagenesis Kit (Agilent Technologies, Santa Clara, CA, USA), we conducted mutagenesis of the seed sequence present in the 3′-UTR to prevent binding of miR-9. The mutations were sequence-verified. Then, the wild-type (WT) and mutated 3′-UTR of MYOCD were cloned into the luciferase reporter plasmid (Promega, Hongkong, China) designated as PGL3-MYOCD-LUC^wt^ and PGL3-MYOCD-LUC^mut^, respectively. For luciferase reporter assay, HEK293 cells were cotransfected with the constitutively active Renilla reniformis luciferase-producing vector pRL, miR-9 or non-targeting pre-miR-negative control and luciferase WT or mutated 3′-UTR vectors for MYOCD using the Siport NeoFX transfection reagent, according to the manufacturer’s instructions. At 24 hrs post-transfection, the cells were lysed, and the relative luciferase expression was measured on a scintillation counter with a dual luciferase reporter system.

### Thymidine (^3^H-TdR) incorporation assay

The same number of PASMCs was seeded into a 96-well culture plate in complete medium. After 24 hrs of synchronous growth, cells were transfected with either miR-9 inhibitor or Myocd according to the manufacturer’s instructions. Then, the cells were incubated with DMEM containing different sera for the indicated time and supplemented with 1 μCi ^3^H-TdR (1 μCi/well) during the last 6 hrs of the treatment period. The incorporation was stopped with cold PBS solution, and 0.25% trypsin was added to digest the cells and separate them from the cell wall. The cells were collected on a glass fibre filter with a multi-head harvester. The cells were washed three times with physiological saline, stabilized with 10% trichloroacetic acid, decolourized with absolute ethanol, and then dried for 30 min. at 80°C, transferred into scintillation fluid, and counted with a liquid scintillation counter (counts/min.).

### CCK-8 assay

Cell proliferation was detected by the Cell Counting Kit-8 assay (CCK-8) (Dojindo, Kumamoto, Japan). 24 hrs after the same number of PASMCs was seeded, cells were transfected with either miR-9 inhibitor or Myocd, and incubated with DMEM containing different sera for the indicated time. At the end of the treatment, 10 μl CCK-8 solution was added to each well, and cells were cultured for 2 hrs more at 37°C. After that, viable cells were detected by measuring the absorbance value at 450 nm using a microplate absorbance reader (Bio-Rad Laboratories, Hercules, CA, USA).

### Statistical analysis

All data were expressed as the mean ± SEM and processed using 17.0 statistical software (IBM, Chicago, IL, USA). Comparisons between groups were carried out using Student’s *t*-test or the Mann–Whitney *U*-test for parametric or non-parametric data, respectively. A *P* < 0.05 was considered to be statistically significant.

## Results

### HPS rat serum up-regulated miR-9 expression, down-regulated myocardin mRNA and protein levels and induced phenotypic modulation in rat PASMCs

**Figure 1:**
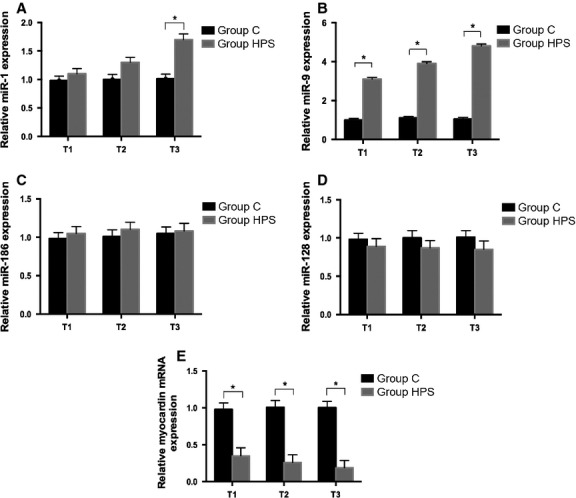
HPS rat serum induces an increase in miR-9 expression level and a decrease in myocardin in cultured PASMCs at each time-point. (A–E) qRT-PCR verification of the selected miRNAs and myocardin expression in cultured PASMCs under HPS rat serum stimulation compared with normal rat serum controls at several time-points. Results were normalized to snRNA U6 levels and expressed as relative fold change. Each data point represents the mean ± SEM of four independent experiments. T1-3: PASMCs were treated with normal rat serum and HPS rat serum for 24 hrs (T1), 48 hrs (T2) and 72 hrs (T3), respectively, *P < 0.05.

**Figure 2:**
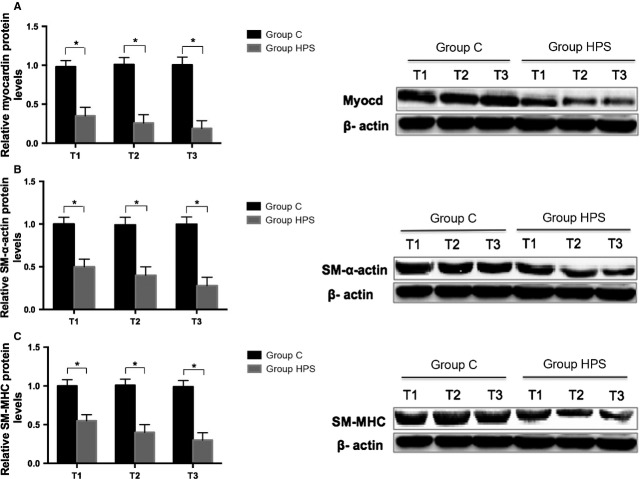
HPS rat serum induces a time-dependent decline in levels of both myocardin protein and the PASMC differentiation markers SM-α-actin and SM-MHC in cultured PASMCs. (A–C) Representative Western blot bands of myocardin and the contractile phenotype markers SM-α-actin and SM-MHC as well as quantification of Western blots are shown. β-actin was used as a loading control. Each data point represents the mean ± SEM of four independent experiments. T1-3: PASMCs were treated with normal rat serum and HPS rat serum for 24 hrs (T1), 48 hrs (T2) and 72 hrs (T3), respectively, *P < 0.05.

Our previous study reported that HPS rat serum induced PASMCs phenotypic modulation and excessive proliferation 4,5. Recently, increasing evidence demonstrates that myocardin plays a direct regulatory role in the phenotypic modulation and proliferation of PASMCs 25,26. Therefore, we began this study with myocardin as the focus molecule and profiled four selected miRNAs, including miR-1, miR-9, miR-128 and miR-186 which were predicted by miRanda (http://www.microrna.org), miRDB (http://www.mirdb.org) and Targetscan (http://www.targetscan.org) to directly target myocardin. Next, cultured PASMCs at 80% confluence were incubated with normal rat serum or HPS rat serum for 24 hrs (T1), 48 hrs (T2) and 72 hrs (T3), respectively, and the selected miRNAs expression were assessed by qRT-PCR. Among these detected miRNAs, miR-9 showed the highest increase (2.8-, 4- and 5.2-fold); miR-1 demonstrated approximately twofold increase, whereas miR-128 and miR-186 showed no significant changes under HPS rat serum stimulation compared with normal rat serum controls at all time-points (Fig. 1A–D). Moreover, the mRNA levels of myocardin decreased significantly at different time-points (Fig. 1E). Interestingly, HPS rat serum induced a time-dependent decline both in the protein levels of myocardin and the PASMC differentiation markers SM-α-actin and SM-MHC in cultured PASMCs (Fig. 2A–C). These results suggest that miR-9 may play a role in HPS rat serum-induced PASMCs phenotypic modulation by negatively regulating myocardin expression.

### Knockdown of miR-9 reverses the HPS rat serum-induced down-regulation of myocardin and differentiation markers in PASMCs, as demonstrated by Western blot analysis

**Figure 3:**
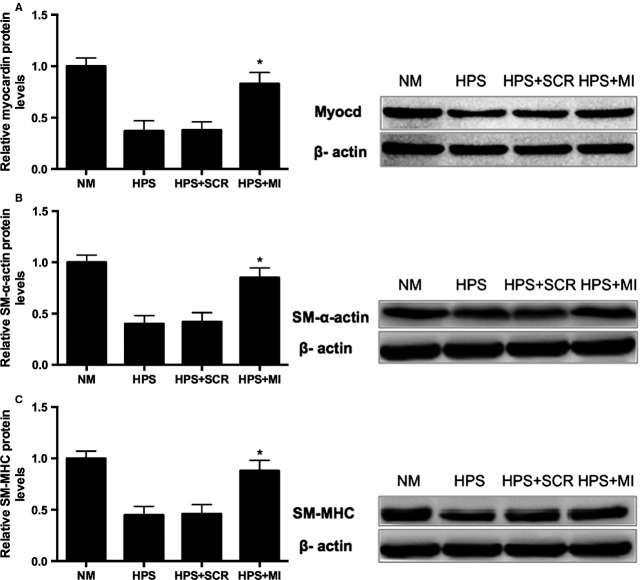
miR-9 plays a direct regulatory role in HPS rat serum-induced PASMC phenotypic modulation *in vitro*. (A–C) Cultured PASMCs were transfected with miR-9 inhibitor (50 nM) or inhibitor control and then treated with HPS rat serum for 48 hrs. Representative Western blot bands and quantitation of myocardin and contractile phenotype markers-SM-α-actin and SM-MHC are shown. Each data point represents the mean ± SEM of four independent experiments. *P < 0.05 compared with group HPS. NM: normal rat serum stimulation; HPS: HPS rat serum stimulation; HPS+SCR: HPS rat serum stimulation + scramble control; HPS+MI: miR-9 inhibitor transfection + HPS rat serum stimulation.

To investigate whether miR-9 functions as a direct regulator of the HPS rat serum-induced PASMC phenotypic switch, miR-9 inhibitor was transfected into cultured PASMCs, and then treated with HPS rat serum for 48 hrs. As demonstrated by Western blot analysis, transfection with miR-9 inhibitor significantly repressed HPS rat serum-induced down-regulation of myocardin and phenotype markers SM-α-actin and SM-MHC (Fig. 3A–C). These findings indicate that miR-9 functions as a potent regulator in HPS rat serum-induced PASMC phenotypic modulation.

### miR-9 efficiently regulates myocardin expression in cultured PASMCs under the condition of HPS rat serum

**Figure 4:**
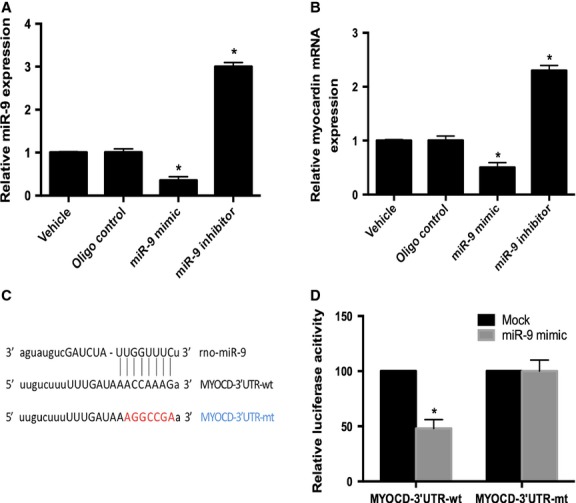
miR-9 directly targets MYOCD 3′-UTR in PASMCs. (A) miR-9 mimic (25 nM) increased and miR-9 inhibitor (50 nM) decreased the expression of miR-9 in cultured PASMCs compared with oligo control. (B) Effect of miR-9 mimic (25 nM) and inhibitor (50 nM) on the expression levels of myocardin in PASMCs stimulated with HPS rat serum for 48 hrs is determined by qRT-PCR. (C) Putative binding site of miR-9 within myocardin 3′-UTR region and its mutated version were shown. (D) miR-9 mimic efficiently inhibited luciferase activity in HEK 293 cells. The inhibitory effect of miR-9 mimic on luciferase activity was abrogated in the mutated reporter group. Each data point represents the mean ± SEM of four independent experiments, *P < 0.05.

To further explore the relationship between miR-9 and myocardin in PASMCs under the condition of HPS rat serum, we transfected cultured PASMCs with miR-9 mimic and inhibitor, respectively, and then treated with HPS rat serum for 48 hrs. According to the qRT-PCR analysis, we observed that the expression of myocardin was efficiently down-regulated by miR-9 mimic and up-regulated by miR-9 inhibitor (Fig. 4A and B). To confirm the effect of miR-9 on myocardin expression was because of its binding to the complementary sites within 3′-UTR of myocardin mRNA, we fused the MYOCD 3′-UTR region, including the predicted miR-9 recognition site, into a luciferase reporter plasmid, designated as PGL3-MYOCD-LUC^wt^. By co-transfecting the miR-9 with this construct into HEK293 cells, we observed that miR-9 mimic effectively repressed luciferase activity, but mutations in the putative binding site eliminated miR-9-mediated repression of luciferase activity (Fig. 4C and D). These results suggest that myocardin serves as a functional target gene of miR-9.

### Myocd mediates the effects of miR-9 on HPS rat serum-induced PASMC phenotypic modulation

**Figure 5:**
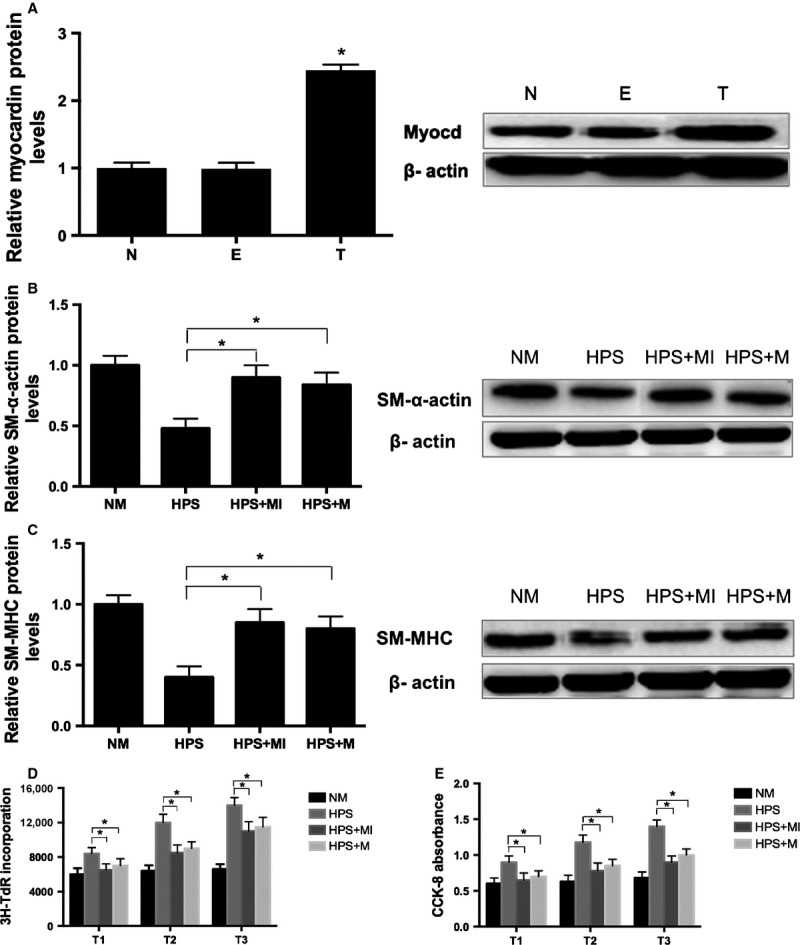
Myocd mediates the effects of miR-9 on HPS rat serum-induced PASMC phenotypic modulation and excessive proliferation. (A) MYOCD protein expression was significantly increased by MYOCD expression plasmid transfection. Representative Western blot bands and mean data generated by densitometry analysis are shown. (B–C) Effect of miR-9 inhibitor and MYOCD overexpression on the expression of contractile proteins SM-α-actin and SM-MHC. Representative Western blot bands and mean data generated by densitometry analysis are shown. (D) DNA synthesis was assessed by 3^H^-TdR incorporation assays. (E) Cell viability was determined by using CCK-8 incorporation. Each data point represents the mean ± SEM of four independent experiments. *P < 0.05. N: Non-transfected group; E: empty vector group; T: transfected group; NM: normal rat serum stimulation; HPS: HPS rat serum stimulation; HPS+MI: miR-9 inhibitor transfection + HPS rat serum stimulation; HPS+M: myocardin expression plasmids transfection + HPS rat serum stimulation.

To further investigate whether the attenuated effect of miR-9 inhibitor on HPS rat serum-induced PASMC phenotypic modulation is associated with myocardin, we applied MYOCD expression plasmids using a transient transfection to overexpress myocardin in PASMCs. After 24 hrs transfection, myocardin levels were significantly increased compared with vehicle and empty vehicle plasmid controls (Fig. 5A). Next, PASMCs were transfected with either miR-9 inhibitor or MYOCD expression plasmids and treated with HPS rat serum for 48 hrs. As shown in Figure 5B and C, inhibition of miR-9 or overexpression of myocardin also repressed HPS rat serum-induced down-regulation of SM-α-actin and SM-MHC. These findings suggest that myocardin not only is a functional target gene of miR-9 but also mediates the effects of miR-9 on HPS rat serum-induced PASMC phenotypic modulation.

### Myocd mediates the effects of miR-9 on HPS rat serum-induced excessive proliferation of PASMCs

The ^3^H-TdR and CCK-8 were performed to further validate the functional role of myocardin in mediating miR-9 effects on HPS rat serum induced PASMCs excessive proliferation. Interestingly, both miR-9 inhibitor and MYOCD expression plasmids efficiently repressed the HPS rat serum-induced increase in ^3^H-TdR incorporation and absorbance of CCK-8 at each time-point (Fig. 5D and E). These findings suggest that myocardin not only is a functional target gene of miR-9 but also mediates the effects of miR-9 on HPS rat serum-induced excessive proliferation of PASMCs.

## Discussion

Hepatopulmonary syndrome, a defective liver-induced lung vascular disorder, occurs as a result of decreased hepatic clearance or increased hepatic production of cytokines and growth factors 27–29. The stimulation of circulating cytokines as well as the development of hypoxaemia induced by intrapulmonary vasodilation contributes to abnormal PASMC phenotypic modulation and proliferation, resulting in PVR 24. Previous studies have identified a sequence of molecular alterations during the onset and progression of HPS, such as nitric oxide, endothelin-1, heme oxygenase-1, carbon monoxide, sex hormones, tumour necrosis factor-α, VEGF as well as some specific chemokines 2,30. However, it is still unclear that which one or two or more components in the serum cause the pulmonary pathology changes until now. We think that understanding the mechanisms of abnormal cell proliferation and differentiation to provide a basis for circumventing the process and creating targeted therapies for diseases associated with vascular remodelling in HPS is another important therapeutic strategy. Our initial research demonstrated that HPS rat serum induced phenotypic modulation and excessive proliferation of PASMCs, which are known to be major pathophysiological characteristics in HPS-associated PVR 4,5. However, the cellular signalling mechanisms involved in HPS-associated PVR remain unknown. Most importantly, whether the regulation of gene expression is an efficient therapeutic target for HPS demands further research. Our present work is the first to reveal that miR-9 and myocardin constitute an axis, which is involved in HPS rat serum-induced PASMC phenotypic modulation and excessive proliferation.

Myocardin is a potent transcriptional co-activator for VSMC-specific gene expression, and strongly inhibits the cell cycle 8. It has been reported that myocardin activity can be regulated by posttranslational modifications, such as phosphorylation and ubiquitination 25, and various environmental cues such as hypoxia, stretch and vascular injury can decrease its expression levels, leading to the PASMC phenotypic switch 26. Although myocardin has been identified as an important regulator of PASMC biology, few upstream signals controlling its expression have been explored. Our recent research has shown that myocardin levels decrease in PASMCs treated with HPS rat serum, and further studies identified myocardin as a direct target of miR-9 and demonstrated its involvement in mediating miR-9 effects on HPS rat serum-induced PASMC phenotypic modulation and proliferation.

MicroRNAs are newly identified modulators of various complicated signalling pathways involved in VSMC biology 31. Liu *et al*. reported that miR-31 is able to promote VSMC proliferation *via* down-regulation of LATS2, which is characterized as a tumour suppressor 32. Recently, miR-133 has been the subject of further functional studies, and results have indicated that it is able to inhibit SMC-specific contractile gene expression by directly targeting several smooth muscle mRNAs as well as SRF 33. It has been reported that miR-21 mediates the effects of the transforming growth factor-β/bone morphogenetic protein (BMP) signalling pathway on VSMC differentiation, and also increases contractile gene expression by repressing PDCD4 34. Wang *et al*.demonstrated that miR-9 inhibits cardiac hypertrophy by suppressing the expression of myocardin in cardiomyocytes 35. In addition, our previous study found that miR-9 plays an important role in hypoxia-induced PASMCs phenotypic modulation and identified myocardin as a downstream molecule 36. However, the role of deregulated microRNAs in PASMC phenotypic modulation and excessive proliferation under the condition of HPS remains unclear.

In the present study, we confirmed the link between HPS rat serum, miR-9 and myocardin. Moreover, we observed that miR-9 is significantly up-regulated under HPS rat serum stimulation and contributes to the HPS rat serum-induced PASMC phenotypic switch and excessive proliferation by targeting myocardin. As was expected, both knockdown of miR-9 and overexpression of myocardin reversed HPS rat serum-induced PASMC phenotypic modulation and excessive proliferation. There is no doubt that our study has shed new light on the understanding of the regulation of HPS machinery by miRNAs.

In conclusion, we described the novel mechanism of miR-9 in regulating HPS rat serum-induced PASMC phenotypic modulation and proliferation *via* the miR-9/myocardin axis. Our findings suggest that up-regulation of miR-9 is critical for the phenotypic modulation of PASMCs in response to HPS rat serum exposure, leading to dedifferentiation of PASMCs by suppressing differentiation genes of PASMCs and enhancing proliferation. Moreover, myocardin is identified as a key molecule in miR-9-mediated effects on the HPS rat serum-induced PASMC phenotypic switch and excessive proliferation. As a result, repression of miR-9 or restoration of myocardin could have important implications for the clinical management of HPS or other proliferative vascular diseases.
